# Clinical Performance of Plasma Aβ_42_/Aβ_40_, p‐tau217 and Neurofilament Light in Sporadic Frontotemporal Dementia Spectrum Disorders with subsequent comparison of pathology

**DOI:** 10.1002/alz.091873

**Published:** 2025-01-09

**Authors:** Binita Rajbanshi, Hilary W. Heuer, Lawren VandeVrede, Peter A. Ljubenkov, Argentina Lario Lago, Igor Prufer Q C Araujo, Adam M. Staffaroni, Leonard Petrucelli, Tania F Gendron, Howard J. Rosen, Brad F. Boeve, Randall J. Bateman, Julio C. Rojas, Adam L. Boxer, ALLFTD Consortium

**Affiliations:** ^1^ Memory and Aging Center, Weill Institute for Neurosciences, University of California San Francisco, San Francisco, CA, USA, San Francisco, CA USA; ^2^ University of California San Francisco (UCSF), San Francisco, CA USA; ^3^ University of California San Francisco, San Francisco, CA USA; ^4^ Memory and Aging Center, Weill Institute for Neurosciences, University of California, San Francisco, San Francisco, CA USA; ^5^ Memory and Aging Center, UCSF Weill Institute for Neurosciences, University of California San Francisco, San Francisco, CA USA; ^6^ Memory and Aging Center, Weill Institute for Neurosciences, University of California San Francisco, San Francisco, CA USA; ^7^ Memory and Aging Center, UCSF Weill Institute for Neurosciences, University of California, San Francisco, San Francisco, CA USA; ^8^ Mayo Clinic, Jacksonville, FL USA; ^9^ University of California, San Francisco, San Francisco, CA USA; ^10^ Department of Neurology, Mayo Clinic, Rochester, MN USA; ^11^ Washington University School of Medicine, St. Louis, MO USA; ^12^ Memory and Aging Center, UCSF Weill Institute for Neurosciences, San Francisco, CA USA

## Abstract

**Background:**

Identification of useful fluid biomarkers is expected to advance frontotemporal dementia spectrum disorders (FTD) care and therapeutic development. The clinical utility of emerging plasma Amyloid, Tau, and Neurodegeneration (ATN) biomarkers in FTD remains unexplored. This study analyzed ATN patterns by sporadic FTD phenotype, based on amyloid beta_1‐42_ (Aβ_1‐42_), amyloid beta_1‐40_ (Aβ_1‐40_), phospho‐tau217 (p‐tau217), and neurofilament (NfL) plasma concentrations.

**Method:**

The present study included 625 participants enrolled in ALLFTD (46% female, median age 61 ± 12 years) for whom biomarker data were available. Plasma Aβ_1‐42_/Aβ_1‐40_ measures were determined by immunoprecipitation‐mass spectrometry whereas plasma p‐tau217 and NfL concentrations were respectively determined by electrochemiluminescence‐ and fluorescence‐based immunoassays. Biomarker concentrations were compared by phenotype and disease severity with ANOVA. Their diagnostic performance was tested with Receiver Operating Characteristic (ROC) curves, and biomarker relationships with measures of clinical severity were tested through linear regressions controlling for age, sex, and APOE genotype.

**Result:**

Higher plasma p‐tau217 and p‐tau217/Aβ_1‐42_, levels were observed in logopenic primary progressive aphasia (lvPPA) and amnestic dementia (AmD) cohorts, compared to other FTD groups. These biomarkers were not associated with disease severity but were affected by APOE4 genotype. High plasma NfL level was observed in fully symptomatic patients compared to controls, and as a function of disease severity. Plasma Aβ_1‐42_/Aβ_1‐40_ level was not different between phenotypes and was not affected by clinical severity. P‐tau217 level, but not Aβ_1‐42_/Aβ_1‐40_ or NfL levels showed high discrimination of lvPPA and AmD from other phenotypes (AUC = 0.87 (95% CI 0.77 – 0.96, p < 0.0001, 73% sensitivity, 92% specificity). The p‐tau217/Aβ_1‐42_ ratio improved this discrimination (AUC = 0.9, 95% CI 0.83 – 1, p < 0.0001, 77% sensitivity, 94% specificity). Plasma Aβ_1‐42_/Aβ_1‐40_, p‐tau217, NfL, p‐tau217/Aβ_1‐42_, p‐tau217/NfL, and (Aβ_1‐42_ x p‐tau217)/NfL were associated with cognitive (MoCA, CVLT), motor (UPDRS), and social behavior (RSMS) performances at baseline.

**Conclusion:**

Plasma p‐tau217/Aβ_1‐42_ identifies, with high specificity, two phenotypes associated with Alzheimer’s disease: lvPPA and AmD. Pending molecular neuroimaging and neuropathological confirmation, this biomarker could be of value to identify primary Alzheimer’s disease pathology or co‐pathology associated with Alzheimer’s disease when sporadic FTD is suspected.

